# Circulating lipids uncover early membrane disruption as a primary event preceding Alzheimer’s disease onset

**DOI:** 10.21203/rs.3.rs-9357482/v1

**Published:** 2026-04-28

**Authors:** Gloria Patricia Cardona-Gómez, Iván Daniel Salomón-Cruz, Nelson David Galvis-Garrido, Laura Alejandra Lozano-Trujillo, Juan Pablo Barbosa-Carvajal, Sara Camila Agudelo-Castrillon, Jorge Montesinos, Taekyung D. Yun, Eliana Henao, Yakeel Quiroz, Kenneth Kosik, Eric Schon, Delfina Larrea, Francisco Lopera, Andrés Villegas, Daniel Camilo Aguirre-Acevedo, Julián D. Arias-Londoño, David Aguillón, Estela Area-Gómez

**Affiliations:** 1 Neurobiology-Neurobank-Clinical Areas. Group of Neuroscience of Antioquia, Faculty of Medicine, Universidad de Antioquia;; 2 CIB, CSIC, Spain;; 3 Boston University, USA;; 4 Biological Science, UCSB;; 5 Columbia University, USA;; 6 Dpt. of System Engineering, Universidad de Antioquia.

**Keywords:** Lipid signature, early biofluid markers, brain-periphery flux, Alzheimeŕs disease, susceptibility and protection

## Abstract

Alzheimer’s disease (AD) is not a single entity but a biologically heterogeneous disorder, with substantial inter-individual variability in clinical presentation, molecular drivers, and disease trajectories. This heterogeneity limits current diagnostic and therapeutic strategies, which largely rely on late-stage protein biomarkers that, while sensitive to prodromal impairment, remain insufficient for primary prevention and patient stratification.

Lipids are fundamental to brain structure and function, yet their role in the earliest stages of AD remains poorly defined. Here, we identify a robust, early-life lipid signature in serum from asymptomatic carriers of the PSEN1-E280A mutation, detectable from childhood. Using latent profile analysis, we resolve distinct lipid states associated with risk and resilience to dementia, modulated by genetic status, sex, and APOE genotype, and supported by concordant changes in circulating protein biomarkers.

Mechanistically, these lipid alterations point to early and sustained disruptions in cholesterol and sphingolipid metabolism, suggesting that impaired lipid turnover is a primary event in AD pathogenesis rather than a downstream consequence. Our findings position circulating lipids as dynamic reporters of disease-relevant cellular pathways and reveal a previously unrecognized metabolic dimension of the presymptomatic phase of AD.

Together, this work reframes AD as a disorder of early metabolic dysregulation and highlights lipidomic profiling as a powerful approach for early detection, risk stratification, and mechanistic insight.

## INTRODUCTION

A definitive diagnosis of Alzheimer’s disease (AD) can only be achieved by postmortem examination of brain tissue and the identification of the two hallmark lesions of the disease: extracellular amyloid-β (aβ) plaques and intracellular neurofibrillary tangles composed of hyperphosphorylated tau protein (pTau). During life, however, clinicians can diagnose “probable AD” with an estimated accuracy of 90–95% by excluding alternative causes of cognitive decline and integrating cognitive assessments, neurological examinations, and neuroimaging data [1]. Early fluid biomarkers based on protein hallmarks are currently used as standard indicators of prodromal cognitive impairment and dementia, along the biological continuum of AD. These include PET-amyloid imaging and cerebrospinal fluid (CSF) Aβ1–42 levels for amyloidosis; imaging biomarkers and CSF tau levels for tauopathy; and neurofilament light chain (NfL) and glial fibrillary acidic protein (GFAP) for neurodegeneration [1]. Recently, phosphorylated forms of tau (p-tau217) in plasma have shown promise as effective peripheral markers for the tauopathy associated with amyloidosis and synaptic loss. However, while these biomarkers are good indicators of neurodegeneration, they are not exclusive of AD pathology [2].

Consequently, there is a critical need to identify early, AD-specific peripheral biomarkers capable of reporting disease risk, tracking progression, and monitoring responses to current and future therapeutic interventions [3]. Such biomarkers would be essential for patient stratification, precision medicine approaches, and the successful development of disease-modifying therapies. In addition to various known and unknown environmental factors, disease heterogeneity is strongly influenced by genetics. To date, mutations and variants in more than 70 genetic loci have been associated with AD risk, implicating diverse biological pathways. Among these, the amyloid precursor protein (APP), Presenilins 1 and 2 (PSEN1 and 2) drive autosomal dominant AD, while the APOE ε4 allele remains the most influential common genetic risk factor for late-onset AD [4]; in contrast, ApoE ε2 is considered AD-protective [5, 6]. Underscoring the role of ApoE in AD, a recently identified Christchurch isoform of ApoE ε3 was shown to confer resistance to AD pathology in carriers of the highly penetrant PSEN1-E280A mutation from the Colombian Familial AD (FAD) cohort [7]. However, the effect of these genes and their clinical impact vary across individuals, focusing the importance of population-specific modifiers [8].

Notably, these and other AD-loci implicate lipoprotein and lipid metabolism as a key component of the AD pathogenesis [9]. Consistent with this, numerous studies have reported early lipid alterations during the course of AD [10, 11]. However, the extent to which these changes contribute to neuronal dysfunction and cognitive decline remains unclear. Clarifying whether peripheral lipid alterations are epiphenomena or reporters of contributing mechanisms is needed for biomarker development in neurodegenerative diseases.

Lipids constitute the most structurally and functionally diverse class of biomolecules, forming highly organized and dynamic membranes that define the identity of each cell type and organelle. Within these membranes, environmental fluctuations can induce lipid phase transitions that alter key biophysical properties, including curvature, thickness, charge, and permeability. Beyond structural roles, lipid organization governs interactions with membrane-associated proteins, modulating their diffusion, clustering, and enzymatic activity [12]. Because lipids operate within tightly interconnected networks, perturbations in their composition can propagate across membranes, transmitting signals between cellular compartments and progressively reshaping both intracellular and extracellular lipid landscapes. In this context, lipids act as highly sensitive reporters of cellular state. However, the biological meaning of most lipid alterations remains poorly understood, contributing to inconsistencies across studies [13].

This challenge is particularly evident in Alzheimer’s disease (AD), where the progressive nature of pathology complicates biomarker discovery. Early intracellular defects arising during presymptomatic stages trigger compensatory responses that may later become maladaptive and are often shared across neurodegenerative conditions. As a result, lipid changes observed at symptomatic stages are difficult to interpret in terms of causality. Resolving this requires the analysis of samples obtained as early as possible, before overt pathology emerges, to capture the initial molecular events driving disease.

To address this, we sought to identify consistent lipidomic alterations in serum from PSEN1 mutation carriers prior to clinical onset and to track their evolution across the lifespan. We analyzed 313 cross-sectional serum samples from carriers and non-carriers of the PSEN1-E280A mutation from the Colombian familial AD kindred [14, 15],, stratified by age and matched by sex. This analysis revealed robust, age-dependent lipid alterations in mutation carriers, with early changes detectable in childhood (6–12 years). Notably, these alterations converge functionally on cholesterol and sphingolipid metabolism, indicating early disruption of lipid homeostasis as a feature of AD pathogenesis.

To further resolve the complexity of lipid variation, we applied latent profile analysis integrating genotype, APOE isoform, sex, sociodemographic factors, comorbidities, and neuropsychological measures. This approach identified five distinct lipidomic states associated with cognitive performance and disease risk in an age-dependent manner, highlighting the context-dependent nature of lipid alterations.

Together, these findings indicate that lipid changes, when interpreted as coordinated states rather than isolated metabolites, provide insight into cellular pathways disrupted during the presymptomatic phase of AD. Our work underscores the need for systems-level approaches and analytical frameworks capable of translating lipidomic complexity into meaningful, high-resolution indicators of cellular function and disease progression.

## RESULTS

### Limitations of Current Protein Biomarkers of Neurodegeneration as Reporters of Clinical Phenotypes

We first aimed to determine the performance of current disease indicators in cross-sectional serum samples spanning presymptomatic to symptomatic stages. For this purpose, we selected 313 cases from the Colombian PSEN1-E280A kindred affected by FAD, obtained from the Biobank of the Group of Neuroscience of Antioquia [15] (Fig. 1a and 1b and Table 1).

Participants were stratified into five age groups: 6–12 years (n = 119, 38%), 13–19 years (n = 48, 15%), 20–30 years (n = 91, 29%), 31–40 years (n = 25, 8%), and ≥41 years (n = 30, 10%). The cohort was balanced by sex (173 females, 55%; 140 males, 45%) and double-blinded with respect to PSEN1-E280A mutation status, comprising carriers (56%) and non-carriers (44%). In addition, cases were classified according to APOE isoforms, including a small number of individuals carrying protective variants APOE Christchurch (APOE ε3Ch/ε3Ch, n = 1; APOE ε3/ε3Ch, n = 3; APOE ε3Ch/ε4, n = 1). Among mutation carriers, only 22% exhibited cognitive impairment, corresponding to 7% of the total cohort (Table 1). Additional demographic and clinical variables, including years of formal education and nutritional status, did not reveal remarkable differences across groups. Furthermore, most participants were free of metabolic, neurological, and neuropsychiatric comorbidities; 19% (n = 25) presented at least one metabolic comorbidity and 24% (n = 30) had neurological or neuropsychiatric conditions (Table 1).

Given prior evidence of age-related increases in several core biomarkers after early adulthood, participants stratified in Table 2, were analyzed into 6–19 years and ≥20 years age groups. Cross-sectional analyses revealed substantial inter-individual variability and extensive overlap between carriers and non-carriers across biomarkers (Fig. 1d – j and Supp. Fig 1). NFL and GFAP showed clear age-associated increases, with higher levels in cases aged ≥20 years, largely independent of genetic background. In contrast, Aβ42 and Aβ40 displayed no consistent age- or carrier-dependent differences, with broadly overlapping distributions across APOE isoforms. Tau-related biomarkers exhibited more nuanced patterns: pTau231 and pTau217 showed a modest tendency toward higher levels in carriers in the ≥20-year group, a trend that was also observed among APOE ε3Ch carriers despite the limited sample size, whereas minimal separation was evident in younger cases; tTau showed limited discrimination across age or genetic groups. Cross-sectional analyses of age-related trajectories further supported these observations, revealing gradual and largely overlapping trends dominated by age- and sex-related effects rather than genotype-specific divergence (Fig. 1, Supp. Fig. 1). Collectively, these data indicate that while current serum indicators of neuronal injury, particularly GFAP, pTau231 and pTau217, may capture aspects of early neuronal damage, they are predominantly influenced by aging or age-related processes and lack sufficient reliability as diagnostic markers or as biomarkers to monitor early disease progression or therapeutic response in this population.

### Age-resolved serum lipidomic reveals an early-adulthood crossover and attenuated lipid remodeling in PSEN1-E280A carriers

To evaluate whether serum lipid changes can serve as reliable biomarkers of disease and identify any potential effect of the PSEN1-E280A mutation on the serum lipid profile, we performed a cross-sectional lipidomic analysis stratified by age, including both sexes. Using serum samples from the cohort participants (Fig. 2a), we isolated and quantified more than 500 lipid species spanning major classes of neutral lipids (sterols and glycerolipids), and polar lipids (glycerophospholipids and sphingolipids) (Fig. 2b).

To obtain a global, age-resolved visualization of lipid-class dynamics, we fit a linear projection model to estimate age-associated trends in lipid-class abundance. To minimize potential confounding by APOE genotype distribution, we performed the primary analysis in APOE ε3/ε3 cases and then repeated the same modeling in the full cohort, including all APOE isoforms. For clarity, lipid classes were displayed in metabolically/structurally related groups: neutral lipids (Fig. 2c, APOE ε3/ε3; Fig. 2d, all isoforms) and sphingolipids (Fig. 2e, APOE ε3/ε3; Fig. 2f, all isoforms), with analogous analyses for phospholipids (Supplementary Fig. 2a, b) and lysophospholipids (Supplementary Fig. 2c, d).

Notably, PSEN mutation carriers exhibited smoother trajectories than non-carriers, particularly at older ages, suggesting reduced inter-individual variability or a stronger influence of shared biological factors, such as the underlying genetic mutation, in this group.

Curve smoothing also revealed a consistent point of inflection before 20 years of age in both groups, suggesting a metabolic transition that may be associated with sexual maturation (Fig. 2 and Supp. Fig. 2), in support of lipidomic profiles being sufficiently sensitive to reflect developmental metabolic changes.

Despite these age-related fluctuations, the major lipid classes glycerolipids, glycerophospholipids, and glycosphingolipids displayed broadly similar overall trends in carriers and non-carriers. However, within specific classes, notable differences emerged. In the neutral lipids category, within the APOE ε3/ε3 subset, non-carriers showed increasing trajectories of free cholesterol (FC) alongside decreasing cholesteryl esters (CE), whereas carriers exhibited the opposite pattern (FC decreasing and CE increasing) (Fig. 2c), suggesting a PSEN1-associated inversion in neutral-lipid remodeling under a homogeneous APOE background. However, when all APOE isoforms were included, both carriers and non-carriers converged on similar age-associated trajectories (FC increasing and CE decreasing) (Fig. 2d). On the other hand, non-carriers displayed increase trajectories in triglycerides (TG) and progressive reductions in acylcarnitine levels (AC) levels, while AC remained comparatively stable in carriers over age (Fig. 2c). Notably, when all isoforms were included in the analysis, the separation in TG trajectories was attenuated, but AC continued with steady levels relative to non-carriers (Fig. 2d), indicating the contribution of lipoprotein metabolism or APOE-related lipid transport mechanisms in the lipid profile of PSEN carriers.

Our results also showed that carriers presented with changes in the trends of the sphingolipid classes analyzed (Fig. 2e and 2f), which were ApoE isoform-independent (Fig. 2f). Specifically, carriers displayed trends of slight increases in ceramides (Cer) and dihydroceramides (dhCer) levels over time compared to non-carriers. The latter also showed sharp progressive reductions in the levels of sphingomyelin (SM) and monohexosylceramide (MhCer), which were not accentuated in carriers of PSEN1-E280A mutations. (Fig. 2e and 2f).

Glycerophospholipids (PLs) and lysoglycerophospholipids (LPLs) analyses revealed changes in the trajectories of some classes in carriers independently of ApoE isoform (Supp. Fig. 2a and 2b). Specifically, carriers presented with steady levels of most abundant PLs and LPLs compared to non-carriers, which in turn displayed progressive decreases in phosphatidylcholine (PC), phosphatidylserine (PS), and phosphatidylethanolamine plasmalogen (PEp) levels, and elevations in those of phosphatidylglycerol (PG) (Supp. Fig. 2a and 2b). Likewise, non-carriers exhibited a progressive reduction in lysophosphatidylcholine (LPC) and lysophosphatidylserine (LPS), while the levels of these classes were essentially unchanged in carriers over time. However, associated to PSEN1 mutation, a divergent cross-sectional pattern for phosphatidylserine (PS) and N-acyl phosphatidylserine (NAPS) classes (Suppl. Fig. 2 a–b).

Taken together, these preliminary observations suggest that lipidomic trajectories could serve as indicators of metabolic fluctuations over time and perhaps reflect the systemic impact of the PSEN1 mutation on lipid metabolism across the lifespan. Moreover, our results indicate that ApoE isoforms can modify the trends of neutral lipid changes, such as FC:CE and TG:AC in carriers, compared to other lipid classes that remained less affected.

### Multivariate analysis of lipidomic data reveals early and age-dependent serum alterations in PSEN1-E280A carriers

To further characterize lipidomic differences associated with PSEN1-E280A carriage independently of ApoE genotype, we applied multivariate analytical approaches commonly used in other omics studies only in those cases with ApoE ε3/ε3, including both sexes (Supp. Fig. 3), thereby minimizing APOE-related heterogeneity while preserving variation by sex. Principal component analysis (PCA) did not clearly discriminate carriers from non-carriers at any age (Supp. Fig. 3a). However, the analysis highlighted FC levels and specific CE species (notably CE 18:2) as major contributors to variance when sexes together per each range of age were examined overtime (Supp. Fig. 3a), consistent with the prominent neutral-lipid shifts captured by the age-resolved models. Complementarily, Partial least squares–discriminant analysis (PLS-DA) identified GM3 species as key discriminators between carriers and non-carriers in the 6–12 yo age group (Supp. Fig. 3 b). PLS-DA also revealed progressive alterations in several phospholipid species, with NAPS emerging as a prominent discriminant class in 20–30 y.o carriers (Supp. Fig. 3 b), indicating that mutation-associated differences are subtle at the global level but become evident within specific lipid subsets and age window.

We next used these selected lipid species fulfilling fold changes 2 and substantially altered in PSEN1 mutation carriers, to construct a preliminary predictive lipid signature using a Random Forest (RF) model (Fig. 3a) using age-stratified feature selection to reflect the age-dependent trajectories observed above. Remarkably, this approach successfully discriminated PSEN1-E280A carriers in an age-dependent manner, achieving areas under the curve (AUC) of 86–93% for FAD cases and 85% for sporadic AD (SAD) cases as an internal comparison (Fig. 3b).

Overall, our data identify specific lipidomic alterations in PSEN1-E280A carriers that are detectable from childhood and evolve dynamically over time. These fluctuations likely reflect ongoing metabolic remodeling, while simultaneously complicating the isolation of mutation-specific effects. Nonetheless, several lipid changes, particularly those involving cholesterol metabolism, exhibited consistent and progressive patterns across sexes beginning early in life. Although these cross-sectional lipid alterations are challenging to interpret mechanistically, they underscore the potential of lipidomics as a sensitive tool for capturing early metabolic disturbances associated with AD–related mutation.

### Univariate analysis of lipidomic data reveals early defects in cholesterol and sphingolipid metabolism in PSEN1-E280A carriers

Lipids act cooperatively to establish and maintain the structural and biophysical properties of cellular and intracellular membranes. Consequently, lipid species within membranes are regulated as an interconnected network to preserve membrane integrity and cellular compartmentalization. Perturbations in one lipid species can therefore propagate to adjacent lipid pools, generating coordinated patterns that provide insight into the underlying biological processes they reflect.

To obtain a comprehensive view of how PSEN1 mutations influence lipid dynamics over time, we analyzed cross-sectional fluctuations in the lipidome of our samples. To minimize confounding effects from different ApoE isoforms, we restricted the primary analysis to individuals homozygous for APOE ε3.

We identified a subset of lipid classes that did not differ between carriers and non-carriers until the mid-twenties, including sphingomyelin (SM) and lysophosphatidylcholine (LPC) (Supp. Fig. 4a). This observation is consistent with pTau217 data indicating that PSEN1-E280A carriers may already exhibit neuronal injury and activation of downstream pathological processes by that age. Notably, these lipid changes were highly variable and strongly influenced by sex. Therefore, we performed sex-stratified analyses focusing on children (6–12 years) and adolescents (13–19 years), who likely represent early stages of mutation-associated cellular dysfunction preceding overt clinical or neuropathological manifestations.

#### Cholesterol metabolism

At the lipid class level, fold-change analyses across age groups in APOE ε3/ε3 cases (Fig. 4a), as well as in analyses including all ApoE isoforms (Supp. Fig. 4b), revealed an increasing trend in total cholesteryl esters (CEs) in carriers beginning at 6–12 years of age. In contrast, free cholesterol (FC) levels were lower in carriers than in controls (Fig. 4a).

Importantly, these early alterations were observed in both males and females, suggesting that sex does not substantially influence PSEN1-related lipid changes during childhood (Fig. 4b). At older ages, however, these patterns became attenuated and more sex-dependent. For example, CE levels remained elevated during adolescence (13–19 years), but at later ages, increases were detectable above noise primarily in male carriers. Cross-sectionally, we observed a progressive rise in FC accompanied by a decline in CE at older ages.

Because coordinated shifts in FC and CE reflect cholesterol partitioning between free and esterified pools, these findings suggest altered cholesterol mobilization and esterification dynamics. Consistently, carriers exhibited an increased FC:CE ratio up to 20 years of age in both sexes (Fig. 4c), even when all APOE isoforms were included (Supp. Fig. 4c). This pattern was not evident at older ages.

Species-level heatmap analyses showed selective enrichment of specific CE molecular species in 6–12-year-old carriers, particularly those containing oleic (C18:1) and linoleic (C18:2) fatty acids, as well as long-chain species such as CE 22:3 (Fig. 4d; Supp. Fig. 4e). These changes were observed in both sexes independently of ApoE genotype. The selective accumulation of these species may indicate increased cholesterol esterification mediated by ACAT or LCAT, suggesting enhanced cholesterol mobilization away from cellular membranes. Elevated levels of some CE species persisted temporally up to 20 years of age, although several species declined in females but not in males at later time points.

Interestingly, despite small numbers, PSEN1 carriers expressing the protective ApoE3ch variant exhibited the opposite pattern—particularly women—highlighting potential sex- and genotype-specific differences in cholesterol handling from early disease stages that may influence individual susceptibility to Alzheimer’s disease (Fig. 4).

#### Sphingolipid metabolism

In children aged 6–12 years, carriers showed marked elevations in several sphingolipid classes, particularly monosialodihexosylganglioside (GM3), independent of sex and ApoE genotype (Fig. 5a; Supp. Fig. 5a). All detected GM3 species were uniformly increased during childhood in carriers. However, at older ages, female carriers exhibited pronounced reductions in GM3 species regardless of ApoE isoform. In males, these decreases were more gradual and primarily evident in APOE ε3 homozygotes.

Other sphingolipid classes, including ceramide (Cer) and dihydroceramide (dhCer), were also elevated during childhood, but predominantly in female carriers (Fig. 5a and 5b), independent of ApoE genotype. In male carriers, alterations were modest and strongly influenced by ApoE isoform.

Species-level analysis in 6–12-year-old female carriers revealed selective increases in long-chain Cer and dhCer species (C26:0 and C26:1)(Fig. 5d), an effect further amplified when all ApoE isoforms were included (Supp. Fig. 5c, 5d). We also observed an overtime decline in sphingomyelin (SM) species in female APOE ε3 carriers, whereas in males this decrease became evident only when all ApoE isoforms were considered. Reductions in SM were mirrored by increases in dihydrosphingomyelin (dhSM), which contains a more saturated sphingoid base that promotes membrane rigidity through cholesterol stabilization. Additionally, female carriers aged 6–12 years displayed reductions in SM and dhSM species containing specific fatty acyl chains implicated in cholesterol solubilization and membrane mobilization.

Collectively, these findings indicate early-life disturbances in sphingolipid flux in female carriers, evolving into sex-specific lipidomic trajectories that are partially modulated by ApoE genotype.

#### Glycerolipid and phospholipid metabolism

Among glycerolipids, children aged 6–12 years showed increased total levels of monoacylglycerides (MGs) and diacylglycerides (DGs), but not triglycerides (TGs) (Fig. 5d). Phosphatidic acid (PA) levels were elevated in both male and female carriers, suggesting enhanced channeling of glycerol intermediates toward phospholipid synthesis.

Within phospholipid classes, marked increases were observed in N-acyl-phosphatidylserine (NAPS) and modest elevations in lysophosphatidylserine (LPS) (Supp. Fig. 5e). Because increased NAPS and LPS are associated with phosphatidylserine (PS) detoxification from cellular membranes, these findings may indicate excess PS accumulation in PSEN1 carriers. Species-level analysis revealed broad elevations in PA and NAPS species in 6–12-year-old carriers of both sexes, with stronger effects in the presence of non-ε3 ApoE isoforms (Supp. Fig. 6a–c) At older ages, female carriers exhibited reductions across several phospholipid classes and species, whereas male carriers showed the opposite trend, suggesting a sex-dependent adaptive remodeling response.

Finally, we did not detect lipidomic alterations in sporadic Alzheimer’s disease (SAD) samples comparable to those observed in PSEN1 mutation carriers, underscoring the distinct metabolic trajectory associated with autosomal dominant disease

### Latent profile analysis of lipid classes identifies endophenotypes associated with clinical and neuropsychological characteristics.

To identify latent subgroups of participants based in the lipidome profiles, we performed Latent Profile Analysis (LPA), as a probabilistic modelling algorithm, which allowed identify five distinct endophenotypic clusters based on differential levels of 16 lipid class<es used as predictive variables to fit a model (Fig. 6a). These lipid classes were selected based on three criteria: a) Low inter-class correlation: A correlation analysis revealed minimal overlap between classes, optimizing the LPA ability to discriminate specific lipids across latent groups. b) Biological criteria, c) Based on Bayesian Information Criterion (BIC) analysis, Akaike Information Criterion, entropy, and minimal class size percentage. We tested models with different number of classes (G = 3–9) and covariance structures were estimated using a Gaussian finite mixture modeling approach implemented in R (version 4.5.1) [16]. Although models with a higher number of classes showed modest improvements in BIC (6 profiles BIC = −5783, 7 profiles BIC = −5766, 8 profiles BIC = −5911), a five class solution was selected as the most parsimonious and interpretable representation of the data while retaining excellent classification quality, based on a high entropy (0.99), acceptable class sizes (min. class %: 5.75%), a BIC of −6101.919 and an average maximum posterior probability of 0.995.

The five clusters showed differential lipid classes profile per cluster (Fig. 6a and 6b). We found a higher correlation of FC in cluster 1, high levels of CE in cluster 2; also, high CE levels, higher GM3 and lowest FC (fc= −1.75) in the cluster 3; around 0 in all lipid classes in the cluster 4; and the high levels of PE, SM, BMP and PC in cluster 5.

Subsequently, those clusters were characterized using clinical, neuropsychological, and sociodemographic data (Table 3). Cluster distribution analysis showed that most of the population was in Cluster 4 (56.64%). Clusters 1 and 2 had the largest percentage of females and cases aged ≥41 years, while the other clusters showed a more even distribution (Fig. 6c and 6d). On the other hand, children (6–12 yo) were primarily grouped in Clusters 4 (46%) and 5 (67%). Regarding genotype, most carriers of the PSEN1-E280A variant were in Clusters 1 and 2 (79% and 82%, respectively). Noncarriers were proportionally more grouped in cluster 4 (54%). However, this cluster also grouped most of the absolute number of carriers in the total samples, most of them younger than 20 y.o. (Fig. 6c and Table 3). Interestingly, protective genotypes carrying at least an ε2 allele (ε2/ε2, ε2/ε3, ε2/ε4) and one ε3*Ch allele (ε3ε3*Ch, ε4 ε3*Ch) were largely concentrated in Cluster 4 with the younger population (Fig. 6e).

Clinically, Clusters 1 and 2 had the largest percentage of symptomatic cases, diagnosed as SCD, MCI, or dementia, including the old woman (72 y.o.) ε3*Ch ε3*Ch in the cluster 1; while Cluster 5 had mostly asymptomatic participants (98%) (Fig. 6c). Interestingly presented 9% of obesity (Fig. 6f, Table 3). Comorbid condition analysis indicated that Clusters 1 and 2 manifested the highest rates of metabolic disorders, such as diabetes and dyslipidemia (Fig. 6f, Table 3) (sum 42% and 20%, respectively), and higher neuropsychiatric condition burden (sum 21% and 28%), respectively (Table 3). In spite of these unique clinical and genetic profiles, biofluid biomarkers were not able to effectively distinguish between clusters (Supp. Fig. 7).

Finally, we also characterized clusters by psychometric variables, such as WISC-IV testing and their sub-items for children and adolescents, CERAD Evocation, Mini-Mental State Examination (MMSE), and Verbal Fluency for adults (Supp. Fig. 8) as described previously [22]. Cluster 1 was composed of cases with lower scores in MMSE, CERAD Word List evocation subitem, and functional classifications using the FAST scale. Regarding children, there was no apparent difference between clusters when described using any of the Composite Indexes for the WISC-IV. Although we notice a lower mean score in the Verbal Comprehension Index for Cluster 1.

## Discussion

In this study, we have used high-resolution mass spectrometry and descriptive statistical analysis, to discriminate lipidomic patterns between carriers of the PSEN1-E280A mutation and noncarriers, which suggests an early disruption of lipid homeostasis that precedes classical other events in Alzheimer’s disease (AD). Although metabolic alterations have traditionally been considered a consequence of neurodegeneration, our findings indicates that lipid metabolism dysregulation may represent an early defect in the disease, detectable even prior to other biomarkers and clinical manifestations.

Changes in specific lipid species have also been suggested as potential biomarkers in AD, yet these findings lack consistency across studies. One of the main limitations in the field is the lack of reproducibility across studies[17] [18] [19].. However, this issue often stems not from technical variability alone, but from an incomplete understanding of what lipid changes actually represent and the multiple factors that influence them. Variables such as sex, genetic background, and environmental context can profoundly shape lipid profiles, complicating the identification of universal biomarkers.

These initial alterations in specific lipids can propagate through functionally linked pathways, triggering compensatory or maladaptive remodeling to preserve membrane integrity. Thus, lipidomic signatures should be interpreted over time and within an integrated and dynamic circular network reflecting coordinated disruptions in membrane structure and cellular signaling rather than single-molecule changes [20]. Possibly, this circular regulatory network that characterizes lipid metabolism contributes to the limited effectiveness of certain multivariate statistical analysis approaches.

Moreover, whether lipidomic serum measurements can capture brain-specific changes also remains poorly understood. To resolve the latter in this study, as PSEN expression is ubiquitous, we assumed that altered lipid pathways are shared between peripheral cells and the central nervous system. Consequently, peripheral alterations may offer indirect insight into broader cellular processes that could also be relevant to neuronal membrane organization and signaling during early stages of pathology.

This also contributes to the variability found in the lipidomics analysis in the literature when studying lipid changes in the blood of symptomatic cases, as more than 15 distinct disease processes operating at different stages of disease progression, impede our understanding of causality and reveal primary drivers of neurodegeneration from downstream consequences of disease [21]- Our work addresses these challenges through longitudinal analyses in carriers of PSEN mutations. These studies allow us to track lipid changes over time, providing a dynamic view that helps distinguish between alterations related to normal aging and those linked to disease processes.

For instance, alterations in sphingomyelin and lysophosphatidylcholine (LPC) levels have been frequently reported in Alzheimer’s disease (AD) [22]. Consistent with previous observations, our data indicate that alterations in these and other lipid classes become evident in carriers after 20 years of age, likely represent relatively late events in disease progression, associated with white matter degeneration and myelin breakdown. In support of this view, defects in SM levels have also been associated to other neurological conditions such as FTD [23] and ALS[24], as a result of disturbances in synaptic integrity, neuroinflammation, and cell death [25, 26] [27] [28]Likewise, increases in LPC and lysophospholipid species have been implicated in AD pathophysiology [29]. These findings may reflect increased phospholipid and membrane remodeling through reacylation pathways [30], often linked to inflammatory signaling and immune activation within the brain [31] [32], and thus, not specific of AD.

Moreover, many of these common lipid alterations appear to be strongly influenced by sex and apolipoprotein E (ApoE) isoform, in agreement with previous studies [33, 34] [35].

Together, these observations suggest that at later stages, many reported lipid disturbances in AD arise from inflammation-driven membrane remodeling processes prominent during later stages of disease. Therefore, these lipid traces might have lower potential as reporters of specific early AD processes, but they can be used to stratify different patients and disease subtypes [36]

The above considerations explained led us to focus on lipid alterations in children up to 12 years of age. In this group, we identified three major changes. First, our analysis revealed alterations in cholesterol metabolism in carriers aged 6–12 years, characterized by reduced levels of free cholesterol (FC) together with increased levels of cholesterol esters (CE). This shift suggests enhanced cholesterol mobilization and esterification, pointing to an early disruption of sterol homeostasis during critical periods of brain maturation.

Dysregulation of cholesterol metabolism and systemic hypercholesterolemia are strongly associated with an increased risk of Alzheimer’s disease (AD)[37, 38]. Beyond APOE, several genes involved in cholesterol regulation—including ABCA1, NSDHL, and CYP46A1—have been genetically linked to AD [39, 40] [41] [42]. In parallel, membrane cholesterol levels critically influence APP processing and presenilin (PSEN1/2) activity[43], further highlighting the central role of cholesterol homeostasis in AD pathophysiology.

Despite this strong association, there is little consensus regarding changes in total cholesterol levels in AD brain tissue. Instead, growing evidence suggests that alterations in cholesterol distribution and turnover—rather than total cholesterol content—are key drivers of disease progression [44].

Increased cholesterol esterification, elevated cholesterol ester (CE) levels, and altered free cholesterol to CE (FC:CE) ratios have frequently been reported in Alzheimer’s disease (AD) patients and across multiple brain cell types, including microglia, astrocytes, and neurons [45] [46] [47]. These processes are largely mediated by acyl-CoA:cholesterol acyltransferase (ACAT) [48] [49]. However, the limited success of ACAT inhibitors in clinical trials suggests that increased cholesterol esterification and efflux may represent compensatory adaptations rather than purely pathogenic processes.

Mechanistically, ACAT activity is closely linked to the formation of mitochondria-associated endoplasmic reticulum membranes (MAMs). These ER subdomains arise in response to elevated cholesterol levels in the ER and promote cholesterol esterification through fatty-acid conjugation [50]. Consistent with the early increase in CE levels observed here, both MAM formation and ACAT activity have been reported to be elevated in AD animal models and in cells derived from AD patients [51]. Although ACAT can utilize several fatty-acid substrates, it preferentially incorporates oleic and linoleic acids, in agreement with our observation of increased CE 18:1 and CE 18:2 species [52]. Nevertheless, a contribution from lecithin–cholesterol acyltransferase (LCAT), whose activity has also been reported to increase in AD, cannot be excluded.

Together, these observations suggest that the lipidomic signatures identified here may reflect increased mobilization of cholesterol from cellular membranes during the earliest phases of disease.

In addition to cholesterol esterification changes, we observed notable changes in some phospholipid classes in carriers aged 6–12 years, particularly N-acylphosphatidylserines (NAPS), a lipid that results from PS binding to a fatty acid. NAPS are highly enriched in both white and gray matter of the human brain and are thought to participate in membrane repair and lipid remodeling. Increased NAPS formation may represent a protective mechanism that removes free fatty acids and phosphatidylserine (PS), the latter being a key signal for apoptotic cell clearance and inflammatory responses [53] [54]. Notably, PS itself is synthesized by phosphatidylserine synthases 1 and 2 (PSS1/2), enzymes that—like ACAT1—localize to MAM domains and have been reported to be upregulated during early disease stages in experimental AD models.

These findings suggest that cells from PSEN mutation carriers may activate early lipid remodeling pathways aimed at preserving membrane integrity under metabolic or structural stress. Consistent with this interpretation, NAPS levels are reduced in brain tissue from cases with late-onset AD, likely reflecting myelin loss and white matter degeneration [55].

We also detected increased levels of phosphatidic acid (PA), a bioactive lipid that plays a central role in membrane dynamics and lipid metabolism. PA functions as a signaling molecule and a key intermediate in the synthesis of triacylglycerols and membrane phospholipids, and its accumulation is often associated with lipid droplet expansion and membrane remodeling. One potential source of increased PA is activation of phospholipase D (PLD), which has been reported to be elevated in AD and to influence amyloid precursor protein (APP) trafficking [56]. Supporting this link, rare variants in the PLD3 gene have been associated with increased susceptibility to AD [57] [58].

A third alteration observed in our cohort was an increase in circulating GM3 ganglioside levels in a subset of PSEN1-E280A carriers, particularly in younger cases. Gangliosides are complex glycosphingolipids derived from ceramide and are highly enriched in the central nervous system, especially at synapses where they organize membrane microdomains that regulate receptor clustering and signal transduction [59] [60]. Different gangliosides exert distinct biological effects: while GM1 promotes neuronal differentiation, elevated GM3 levels can inhibit neuronal growth and differentiation [60]. Ganglioside balance also influences cellular metabolism and signaling pathways, including insulin and leptin signaling [61].

Elevated GM3 levels have been reported in AD and other neurodegenerative conditions. Recent work further suggests that GM3 accumulation in microglia contributes to impaired amyloid-β clearance and promotes neuroinflammatory responses during disease progression [62] [63] [64] [21].

Taken together, our results reveal early lipidomic alterations in PSEN mutation carriers that converge on a common theme of membrane remodeling and lipid trafficking dysregulation. Increased cholesterol mobilization, enhanced PS remodeling, and changes in sphingolipid signaling may collectively reflect early cellular attempts to maintain membrane homeostasis in the face of metabolic stress. These processes are likely to affect distinct cell types differently, potentially contributing to early synaptic vulnerability in neurons and inflammatory activation in glial cells. Notably, our observations are consistent with reports of early MAM upregulation and its downstream effects on lipid metabolism, supporting the idea that MAM dysfunction may represent a central hub linking lipid dysregulation to Alzheimer’s disease pathogenesis [65]. Although serum data alone cannot establish a direct link to specific neuronal processes, it raises the possibility that these lipids may serve as a peripheral marker of neurobiological dysfunction. Previous studies have also observed that lipid data can classify different AD subtypes linked to specific events in the disease [36].

However, a larger number of cases would be necessary to validate some of these lipid alterations as AD biomarkers.

Despite this limitation, our latent profile analysis (LPA) was able to cluster a high proportion of carriers of the E280A variant exhibiting profound disruption in cholesterol metabolism, and a high proportion of carriers of the E280A variant, predominantly adult women, many of whom already exhibit cognitive symptoms.

On the other hand, cluster 5 gathers the youngest participants mostly non-carriers of the PSEN1-E280A mutation and shows a high frequency of the protective APOE ε2 allele, generally representing a “healthier” non-AD clinical profile.

Our study has several limitations. The number of cases in some groups is small, and technical constraints may preferentially enhance the solubilization of certain lipid classes over others; therefore, we cannot exclude the presence of alterations in additional lipid species that were not detected. Moreover, the functional roles of many lipid species in biological membranes remain poorly understood, making it difficult to fully interpret how their alteration contributes to cell dysfunction. It is also possible that PSEN mutation carriers already exhibit lipid alterations secondary to defects in key processes such as myelination and neuronal development, which may not be present in other genetic backgrounds. Finally, observed sexual differences may reflect an increased susceptibility to disruptions in cerebral lipid metabolism in females; however, these findings do not necessarily imply causality, but the influence of hormonal factors.

Taken together, our findings indicate that peripheral lipid profiles capture early biological alterations during the presymptomatic phase of Alzheimer’s disease in PSEN1-E280A mutation carriers. By leveraging a longitudinal framework, we begin to disentangle cause from consequence, revealing that disruptions in cholesterol metabolism arise remarkably early—potentially during childhood in genetically predisposed individuals. Importantly, these changes do not reflect isolated alterations in individual lipid species, but rather a broader dysfunction in lipid turnover. Our data further highlight lipidomics as a powerful source of early disease reporters, capable of providing insight into the mechanisms underlying disease initiation. However, the strong influence of factors such as sex and APOE genotype argues against the existence of one unique single lipid biomarkers, and instead supports a model in which coordinated lipid states more accurately reflect underlying biological processes. Advancing such systems-level approaches will be essential to resolve the functional roles of lipid alterations and to refine our understanding of the molecular basis of Alzheimer’s disease.

## MATERIALS AND METHODS

### Study participants

We retrospectively analyzed a cross-sectional dataset of 316 individuals selected from the historical Colombian PSEN1-E280A kindred registry. This registry, maintained by the Grupo de Neurociencias de Antioquia (GNA), encompasses over three decades of longitudinal follow-up for approximately 2,700 individuals. Participants were selected based on the availability of serum samples, comprehensive clinical assessments conducted between 2000 and 2020, and genetically confirmed PSEN1-E280A status. Not a priori sample-size calculation was performed. Three individuals were excluded due to confounding neurodegenerative or neurodevelopmental comorbidities.

To capture distinct neurodevelopmental and disease stages, participants were stratified into five age groups: childhood (6–12 years), adolescence (13–19 years), early adulthood (20–30 years), mid-adulthood (31–40 years), and late adulthood (≥41 years). APOE genotype was characterized, and PSEN1 carrier status was blinded to investigators (labeled as Groups A and B) during lipidomic analysis. The study adhered to ethical standards approved by the University of Antioquia’s Institutional Bioethics Review Committee, with written informed consent obtained from all participants or legal guardians (see Supplementary Table 1 for baseline characteristics).

### Clinical and sociodemographic variables

Clinical and sociodemographic data were accrued via semi-structured interviews conducted by neurologists or general practitioners. Assessments encompassed socioeconomic status, educational attainment, medical history, and a comprehensive neurological examination. Diagnostic staging followed established criteria: Subjective Cognitive Decline (SCD) was defined by the presence of self-reported cognitive complaints in the absence of objective impairment on age- and education-adjusted neuropsychological assessments [66]. Mild Cognitive Impairment (MCI) and dementia were diagnosed in accordance with DSM-5 guidelines. Functional independence was quantified using the Lawton–Brody Instrumental Activities of Daily Living (IADL) Scale and the Barthel Index. Nutritional status was indexed by Body Mass Index (BMI), and comorbidities were verified through medical records and caregiver reports.

### Neuropsychological assessments

Cognitive performance in adults was assessed using the Colombian validation of the Consortium to Establish a Registry for Alzheimer’s Disease (CERAD) battery ^5^, covering memory, attention, language, praxis, and executive functions. Disease progression and functional severity were staged using the Lawton–Brody and Barthel indices, the Functional Assessment Staging Test (FAST), and the Global Deterioration Scale (GDS)-Affective symptoms were screened using the Geriatric Depression Scale.

Pediatric participants (<18 years) followed the GNA’s standardized protocol, incorporating the Evaluación Neuropsicológica Infantil (ENI) and the Behavior Assessment System for Children (BASC-2) for behavioral profiling. General intellectual functioning was quantified via the Wechsler Intelligence Scale for Children (WISC-IV). In the absence of specific local norms at the time of assessment, scores were derived using the Chilean standardization, selected as the most demographically and linguistically proximal reference available for the Colombian population.

### Lipidomic analyses

Serum samples (0.5 mL) from the Biobank of the Grupo de Neurociencias de Antioquia (GNA) were processed individually using the established Folch liquid-liquid extraction method, as in [24, 67]. Lipids were extracted with a chloroform:methanol mixture (2:1 v/v) containing 0.005% butylated hydroxytoluene (BHT) as an antioxidant. Phase separation was achieved by homogenization, followed by the addition of 1 mL of 0.9% NaCl and centrifugation (3000 rpm, 3 min). The resulting organic phase was collected, evaporated to dryness under nitrogen, and lyophilized to ensure complete removal of residual moisture. Dried lipid extracts were subsequently subjected to mass spectrometric profiling. Quantitative lipidomic profiling was conducted via automated electrospray ionization tandem mass spectrometry (ESI–MS/MS) on an API 4000^™^/QTRAP 4000 platform (AB Sciex) at the Kansas Lipidomics Research Center (KLRC), following their standardized protocols ^14^. Each sample was spiked with internal standards corresponding to major lipid classes, including LPG(14:0), LPG(18:0), PG(14:0/14:0), LPE(14:0), LPE(18:0), LPC(13:0), LPC(19:0), PC(12:0/12:0), PC(24:1/24:1), LPA(14:0), LPA(18:0), PA(14:0), PA(20:0/20:0), PS(14:0/14:0), PS(20:0/20:0), PI(16:0/18:0), and PI(18:0/18:0). Lipid molecular species were identified based on their mass-to-charge ($m/z$) ratios and characteristic fragment ions. Quantification relative to internal standards was performed using the LipidomeDB Data Calculation Environment (http://lipidome.bcf.ku.edu:8080/Lipidomics/index.jsp). A total of 34 distinct lipid classes were quantitatively identified, with individual species defined by their total carbon number and degree of unsaturation. Lipid concentrations were normalized to molar percentages (%mol) across all detected species within each sample.

### Statistical analysis

Statistical analyses were performed transversely within a retrospective design framework. Baseline characteristics were summarized as n (%) for categorical variables and mean ± SD for continuous variables. Group differences were assessed using Fisher’s exact test (categorical) and the Kruskal–Walli’s test (continuous), given that the Shapiro–Wilk test indicated a predominant non-Gaussian distribution across continuous variables. Lipid concentrations were calculated as the sum of total moles per class and normalized to molar percentage (%mol). For robust lipidomic analyses, only species detected in ≥80% of samples were included. To visualize lipidomic remodeling, heatmaps were generated using log2 fold-change (FC) values, referencing non-carriers harboring the APOE 3/3 genotype as the control group. For multiple comparisons, Kruskal–Wallis was followed by Dunn’s post hoc test, with p-values adjusted using the Benjamini–Hochberg False Discovery Rate (FDR) correction. Multivariate modeling captured global lipidomic variance. Principal Component Analysis (PCA) was employed as an unsupervised approach, and Partial Least Squares–Discriminant Analysis (PLS–DA) was implemented as a supervised dimensionality reduction method. Model quality was assessed via leave-one-out cross validation, with cluster separation defined by 95% confidence ellipsoids. Discriminant variables were identified using the Variable Importance in Projection (VIP) and Significance Multivariate Correlation (sMC) indices over the components provided by PLS-DA to minimize non-informative variance. Receiver Operating Characteristic (ROC) curves and Pearson’s correlation matrices were constructed to evaluate predictive accuracy and interclass dependencies, respectively.

Post-mortem brain tissue datasets were analyzed separately using GraphPad Prism v8.0. Comparisons across diagnostic categories (Healthy, sporadic AD, and familial AD) and sex-stratified analyses utilized one-way ANOVA and Tukey’s post hoc tests, or appropriate non-parametric equivalents. Results are expressed as mean ± SEM, with statistical significance set at p < 0.05. Significance thresholds were defined as: p < 0.05 (*), p < 0.01 (**), and p < 0.001 (***).

### Latent Profile Analysis

Latent Profile Analysis (LPA) was employed as a model-based probabilistic clustering approach to identify unobserved (latent) subgroups within the lipidomic dataset. This method assumes that population heterogeneity can be represented by a finite mixture of continuous multivariate distributions, facilitating the detection of biologically meaningful endophenotypes not apparent through manifest variables alone. The LPA input matrix consisted exclusively of quantitative lipid species measurements, ensuring that clustering reflected intrinsic lipidomic variance rather than external covariates (genetic status, sociodemographic, or clinical scores were excluded from this analysis). Prior to model estimation, lipid classes included in the LPA were selected based on pairwise correlation analyses and inspection of distribution matrices, ensuring that only species with coherent covariance structures and non-redundant signal patterns contributed to latent class formation (Supp. Fig. 1). Models specifying one to nine latent classes were estimated using Gaussian finite mixture modeling assumptions in R (v4.4.1) within the RStudio environment (v2025.05.1), utilizing the Mclust package (version 6.1.1).

Model selection was guided by the Bayesian Information Criterion (BIC), the Akaike Information Criterion (AIC), and entropy values, jointly optimizing model parsimony and the quality of the latent-profile clustering. The optimal number of profiles was determined as the model minimizing BIC while maintaining high entropy, which is indicative of stable cluster assignment probabilities. Beyond statistical fit indices, biological interpretability was incorporated as an essential selection criterion. This refinement prioritized profiles that captured coherent lipidomic trajectories and exhibited neurobiological relevance, ensuring class discrimination was meaningful. The resulting latent profiles delineate subgroups with internally homogeneous lipid distributions and distinct interclass separation patterns. This data-driven stratification provides a framework to evaluate whether lipidomic architecture can uncover early endophenotypes associated with Alzheimer’s disease (AD) risk, enabling the identification of metabolic substructures potentially reflecting early pathophysiological divergence for improved disease stratification and precision-targeted interventions.

### Simoa Biomarker Quantification and Quality Control

Serum concentrations of neurofilament light chain (NfL), glial fibrillary acidic protein (GFAP), amyloid-β40 (Aβ40), amyloid-β42 (Aβ42), total tau (t-tau), and phosphorylated tau at threonine 231 (pTau231) and threonine 217 (pTau217) were measured using the SR-X Ultra-Sensitive Biomarker Detection System (Quanterix, Lexington, MA, USA) based on Single Molecule Array (Simoa) technology (Simoa^®^ Technology | Quanterix). A total of N = 173 participants with available serum were included in this analysis (see Supplementary Table 2 for demographic characteristics and stratification by genotype, age group, and cognitive status). Analyses were performed according to manufacturer instructions using the following kits: Simoa Neurology 2-Plex B, Human Neurology 3-Plex A (N3PA), pTau-231 Advantage, and ALZpath pTau-217 Care Advantage.

The NfL–GFAP, Aβ40–Aβ42–t-tau, and pTau217 assays were conducted using a two-step protocol, whereas pTau231 followed a three-step protocol. Calibrators and serum samples were run in duplicate and diluted 1:4, except for pTau217 (1:3). Concentrations were derived from 4-parameter logistic (4-PL) calibration curves.

Each assay run includes low- and high-level quality controls (QC1 and QC2) to monitor inter-assay variability. Plate-specific correction factors were calculated from the ratio between the overall geometric mean of QC values and the observed value for each plate. Intra-assay and inter-assay coefficients of variation were maintained below 15% and 20%, respectively. When QC1 and QC2 deviated in opposite directions, a two-point anchoring correction based on the regression of expected versus observed values was applied. Measurements below the lower limit of quantification (LLOQ) were excluded from further analyses.

### Statistical Analysis for Simoa Data

Statistical analyses were performed in R (v4.4.1) using RStudio (version 2025.05.1). Continuous variables were summarized as mean ± standard deviation or median (IQR), depending on distribution, and categorical variables as counts and percentages. Data cleaning and descriptive statistics were generated using the tidyverse suite.

Associations between serum biomarker concentrations and age were examined using linear models stratified by genetic status (PSEN1 mutation carriers vs. non-carriers) or demographic subgroups when appropriate. Group comparisons were conducted using the Mann–Whitney U test for two groups or the Kruskal–Wallis test for multiple age strata, followed by Dunn’s post hoc testing with Benjamini–Hochberg false discovery rate correction. Data visualizations were generated using ggplot2, and final layouts were assembled with the patchwork package[68].

## Supplementary Material

1

## Figures and Tables

**Figure 1.** Demographic characterization and serum neurodegeneration–associated protein profile of the PSEN1-E280A cohort. (a) Distribution of the study population by sex and PSEN1-E280A carrier status. (b) Age distribution of participants. (c) Workflow schematic analysis the biomarker of neurodegeneration-associated proteins quantified using SIMOA technology. (d–j) Violin plots showing serum protein biomarker distributions stratified by age group (6–19 and ≥20 years) and sex: Aβ42 (d), Aβ40 (e), NfL (f), GFAP (g), pTau231 (h), pTau217 (i), and total tau (j). In all violin plots, A denotes PSEN1-E280A non-carriers and B denotes PSEN1-E280A carriers. Asterisks indicate statistically significant differences between groups (*P < 0.05; **P < 0.01; ***P < 0.001). Statistical comparisons were performed using Student’s t-test for parametric data and the Mann–Whitney U test for non-parametric data. Statistical analyses were restricted to individuals with the APOE ε3/ε3 genotype. Sample sizes represent the total number of serum samples analyzed for each biomarker, including females and males, carriers and non-carriers, as follows: Aβ42 (n = 116), Aβ40 (n = 107), NfL (n = 153), GFAP (n = 127), pTau231 (n = 108), pTau217 (n = 173), and total tau (n = 92).

**Figure 2.** Age-associated trajectories of serum lipid classes assessed by mass spectrometry. (a) Workflow schematic overview of the serum lipidomic based on mass spectrometry analysis. (b) Summary of lipid classes analyzed, grouped into neutral lipids (sterols and glycerides) and polar lipids (glycerophospholipids and sphingolipids). (c–d) Age-associated trends of neutral lipid classes modeled using linear projection (APOE ε3/ε3 in c; all APOE isoforms in d). (e–f) Age-associated trends of sphingolipid classes modeled using linear projection (APOE ε3/ε3 in e; all APOE isoforms in f). The total number of serum samples included in the lipidomic analyses was n = 313, including females and males; analyses are shown for the APOE ε3/ε3 subset and for the full cohort including all APOE isoforms.

**Figure 3.** Age-associated lipidomic alterations across the lifespan in APOE ε3/ε3 individuals. (a) Graphs displaying by log_2_ fold changes and significantly altered lipid species across age groups (6–12, 13–19, 20–30, 31–40, and ≥41 years, and sporadic Alzheimer’s disease (SAD), 50–90 years). Analyses were restricted to APOE ε3/ε3 individuals and collapsed across sex within each age group (females and males pooled). (b) Receiver operating characteristic (ROC) curves illustrating the discriminatory performance of age-associated lipid signatures across age groups, evaluated using leave-one-out cross-validation (LOOCV). Sample sizes (APOE ε3/ε3; females and males pooled) were: 6–12 years (n = 73), 13–19 years (n = 34), 20–30 years (n = 72), 31–40 years (n = 16), and ≥41 years (n = 13).

**Figure 4.** Sex and age-stratified lipid-class remodeling highlights early cholesterol alterations in APOE ε3/ε3 individuals (a) Heatmap of log_2_ fold changes at the lipid-class level, stratified by age group and sex. In females, the APOE3ch/3ch PSEN1-E280A carrier is shown separately (3*3*). (b) Linear graph representation and (c) violin plots showing class-level distributions of free cholesterol (FC) and cholesteryl esters (CE) (% mol), stratified by age group (6–12, 13–19, 20–30, and ≥30 years) and sex. In all violin plots, A denotes PSEN1-E280A non-carriers and B denotes PSEN1-E280A carriers. (A, non-carriers; B, carriers). (d) Linear graph representation of longitudinal changes in cholesteryl ester species bound to oleic (CE18:1) and linoleic (CE 18:2) acids. Asterisks indicate statistically significant differences (*P < 0.05; **P < 0.01; ***P < 0.001). Statistical analyses were restricted to APOE ε3/ε3 individuals. Sample sizes are reported in the Methods.

**Figure 5.** Sex and age-stratified lipid-class remodeling highlights early sphinolipid alterations in APOE ε3/ε3 individuals. (a) Violin plots of sphingolipid classes (ceramides (Cer), dihydroceramides (dhCer), sphingomyelins (SM), dihydrosphingomyelins (dhSM), and monosialodihexosylganglioside (GM3) in the 6–12-year age group, stratified by sex. (b) Linear graphs representing the longitudinal changes the indicated sphingolipid classes. (c) Representation of the progressive reductions in the indicated SM species in female carriers. (d) Violin plots of glycerolipid mono-, di- and triglycerides classes; and (e) of phospholipid classes phosphatidic acid (PA) and N-acylphosphatidylserine (NAPS) in the 6–12-year age group, stratified by sex. Asterisks indicate statistically significant differences (*P < 0.05; **P < 0.01; ***P < 0.001). Statistical analyses were restricted to APOE ε3/ε3 individuals. Sample sizes are reported in the Methods.

**Figure 6.** Latent lipidomic organization defines developmental and clinical endophenotypes aligned with lifespan inflection points. (a) Latent Profile Analysis (LPA) identifying five lipidomic clusters derived from 16 representative lipid classes. (b) Bubble plot illustrating inter-cluster similarity based on correlations among lipid classes. (c) Distribution of PSEN1-E280A non-carriers (A) and carriers (B) across the five lipid-defined clusters, stratified by sex. (d) Age distribution of participants within each lipidomic cluster. (e) Distribution of APOE isoforms across clusters; APOE Christchurch variants are indicated by an asterisk (*). (f) Distribution of metabolic comorbidities across lipid-defined clusters, including diabetes (DIA), dyslipidemia (DLP), and obesity (OBS). (g, h) Neuropsychological performance across lipid-defined clusters. Children and adolescents (6–16 years) were evaluated using WISC-IV composite indices (g), whereas adults (≥18 years) were assessed using the MMSE, CERAD Word List Recall, and Semantic Verbal Fluency tests (h). In all violin plots, A denotes PSEN1-E280A non-carriers and B denotes carriers. All cognitive measures were normalized to z-scores and integrated into a global neuroperformance index.

## Data Availability

Mass spectrometry–based lipidomics data have been deposited in the Nbiol-GNA-UdeA/E280A-Serum-Lipidomics-Repository-GNA-Cohort: E280A Serum partner repository (dataset identifier: [![DOI](https://zenodo.org/records/17693328)] (https://doi.org/10.5281/zenodo.17693327). This repository contains serum lipidomic profiles from 313 cases of the Colombian PSEN1-E280A kindred, curated by the Grupo de Neurociencias de Antioquia (GNA), spanning ages 6–≥41 years and including APOE genotypes. All processed data supporting the findings of this study are available in the Supplementary Information and Source Data files. All reagents used in this study are commercially available, and catalog numbers are listed in the Reagents Table (Supp. Table 4 and 5). Source data are provided with this paper.
