# Supplementary material for: Circulating lipids uncover early membrane disruption as a primary event preceding Alzheimer’s disease onset
Source: Res Sq. 2026 Apr 28:rs.3.rs-9357482. Preprint. [Version 1] doi: 10.21203/rs.3.rs-9357482/v1 (PMC13142613; doi:10.21203/rs.3.rs-9357482/v1)
Supplement: 1 [file NIHPPRS9357482V1-supplement-1.pdf]

Supplementary figure legends

Supplementary Figure 1. Age-associated serum SIMOA biomarker trajectories stratified by sex and PSEN1 E280A carrier status. (a–e) Age-associated trajectories of serum SIMOA biomarkers (pg mL^{-1}), displayed separately for females and males and stratified by PSEN1 E280A carriers and non-carriers. Biomarkers include Aβ40 (a), Aβ42 (b), pTau217 (c), pTau231 (d), and total tau (e). All SIMOA biomarkers (Aβ40, Aβ42, NfL, GFAP, pTau231, pTau217, and total tau) were analyzed across age using all APOE isoforms represented in the lipidomic subset, complementing the analyses shown in Figure 1. Sample sizes represent the total number of serum samples analyzed per biomarker: Aβ42 (n = 116), Aβ40 (n = 107), NfL (n = 153), GFAP (n = 127), pTau231 (n = 108), pTau217 (n = 173), and total tau (n = 92).

Supplementary Figure 2. Age-associated trajectories of serum phospholipid and lysophospholipid classes modeled using linear projection. (a–b) Age-associated trends of phospholipid classes modeled using linear projection (APOE ε3/ε3 in a; all APOE isoforms in b). (c–d) Age-associated trends of lysophospholipid classes modeled using linear projection (APOE ε3/ε3 in c; all APOE isoforms in d). The total number of serum samples analyzed was n = 316, including females and males and all APOE isoforms.

Supplementary Figure 3. Multivariate analysis of serum lipidomic in APOE ε3/ε3 individuals across age groups. (a) Principal component analysis (PCA) by age group showing score plots (sample clouds) and corresponding loading plots. (b) Partial least squares–discriminant analysis (PLS-DA) by age group showing score plots (sample clouds) and corresponding loading plots. Sample sizes (APOE ε3/ε3; females and males pooled) were: 6–12 years (n = 73), 13–19 years (n = 34), 20–30 years (n = 72), 31–40 years (n = 16), and ≥41 years (n = 13).

Supplementary Figure 4. APOE isoform distribution and APOE-stratified lipid-class and lipid-species fold-change heatmaps. (a) Representation of the indicated lipid classes in longitudinal samples from carriers and non-carriers APOε3/ε3, before and after stratifying by sex. (b) Heatmap illustrating age-dependent trajectories of lipid classes across APOE genotypes, stratified by sex and referenced to age- and sex-matched APOE isoforms individuals different to APOε3/ε3. The color scale represents $\log_2$ fold-change relative to controls (blue, decreased; white, no change; red, increased). Panels A and B correspond to PSEN1-E280A non-carriers and carriers, respectively, for each APOE isoform shown. (c) representation of the progression in FC:CE ratio. (d) Heatmap of $\log_2$ fold changes at the lipid-class level, stratified by age group and sex, including

all APOE isoforms. Asterisks indicate classes showing statistically significant differences between carriers and non-carriers. (e) Heatmap of $\log_2$ fold changes for cholesteryl ester (CE) species, stratified by age group and sex, restricted to APOE ε3/ε3 individuals or (f) including all APOE isoforms. In females, the APOE3ch/3ch PSEN1-E280A carrier is shown separately (3*3*). Asterisks indicate species showing statistically significant differences between carriers and non-carriers.

Supplementary Figure 5. Age- and sex-stratified fold-changes in sphingolipids. (a) Representation of the longitudinal changes in GM3· levels. (b) Heatmap of $\log_2$ fold changes for ceramide (Cer) and dihydroceramide (dhCer) species, (c) GM3 and (d) sphingomyelin (SM) and dihydrosphingomyelin (dhSM) stratified by age group and sex, restricted to APOE ε3/ε3 individuals or including all APOE isoforms. In females, the APOE3ch/3ch PSEN1-E280A carrier is shown separately (3*3*). (e) Representation of the longitudinal changes in the indicated phospholipid and (f) glycerolipids classes before and after stratifying by sex. Asterisks indicate species showing statistically significant differences between carriers and non-carriers.

Supplementary Figure 6. Age- and sex-stratified fold-change heatmaps of phospholipid and glycerolipid species in APOE ε3/ε3 individuals Heatmap of $\log_2$ fold changes for phosphatidic (a) acid (PA), (b) PE and (c) NAPS, stratified by age group and sex, restricted to APOE ε3/ε3 individuals. In females, the APOE3ch/3ch PSEN1-E280A carrier is shown separately (33). Asterisks indicate species showing statistically significant differences between carriers and non-carriers. Asterisks indicate species showing statistically significant differences between carriers and non-carriers. In females, the APOE3ch/3ch PSEN1-E280A carrier is shown separately (3*3*). Asterisks indicate species showing statistically significant differences between carriers and non-carriers.

Supplementary Figure 6. Distribution of SIMOA-derived protein biomarkers across lipidomic LPA clusters. Serum SIMOA biomarker concentrations (pg mL^{-1}) were normalized and expressed as percentages relative to the maximum observed value for each analyte. Panels depict the distribution of individual biomarkers across the five clusters defined by latent profile analysis (LPA): (a) neurofilament light chain (NfL), (b) glial fibrillary acidic protein (GFAP), (c) Aβ40, (d) Aβ42, (e) total tau, (f) pTau231, and (g) pTau217.

Supplementary Figure 7. Latent lipidomic clusters align with neuropsychological performance across the lifespan. Summary of cognitive performance across ages using standardized neuropsychological assessments. In children and adolescents (6–19 years), performance is shown for WISC-IV composite indices, including the Verbal Comprehension Index (VCI), Perceptual Reasoning Index (PRI), Working Memory Index (WMI), and Processing Speed Index (PSI). In adults (≥20 years), cognitive performance is assessed using the CERAD Word List Recall score, the Mini-Mental State Examination (MMSE), and the Semantic Verbal Fluency test. Column (a) displays violin plots of cognitive performance stratified by PSEN1 E280A carrier status (A, non-carriers; B, carriers). Column (b) show the distribution of cognitive scores across lipidomic clusters identified by latent profile analysis (LPA). Column (c) Compare groups A and B per cluster: cluster 1 (pink), cluster 2 (yellow), cluster 3 (green), cluster 4 (blue), and cluster 5 (purple). All cognitive measures were standardized and expressed as mean z-scores to enable direct comparison across age groups and cognitive domains.
